# The Value of Meteorological Data in Optimizing the Pattern of Physical Load—A Forecast Model of Rowing Pacing Strategy

**DOI:** 10.3390/ijerph19010320

**Published:** 2021-12-29

**Authors:** Tian Yan, Xiaodong Zhu, Xuesong Ding, Liming Chen

**Affiliations:** 1 Division of Sports Science and Physical Education, North China University of Science and Technology, Tangshan 063210, China; Yantianhaha@ncst.edu.cn; 2 Division of Sports Science and Physical Education, Tsinghua University, Beijing 100084, China; zhuxiaodong@tsinghua.edu.cn; 3 School of Economics and Management, Beijing University of Posts and Telecommunications, Beijing 100876, China; dingxuesong2000@bupt.edu.cn; 4 Division of Sports Science and Physical Education, Tangshan Polytechnic College, Tangshan 063299, China

**Keywords:** rowing, meteorological data, pacing strategy, physical load, forecast

## Abstract

Mastering the information of arena environment is the premise for athletes to optimize their patterns of physical load. Therefore, improving the forecast accuracy of the arena conditions is an urgent task in competitive sports. This paper excavates the meteorological features that have great influence on outdoor events such as rowing and their influence on the pacing strategy. We selected the meteorological data of Tokyo from 1979 to 2020 to forecast the meteorology during the Tokyo 2021 Olympic Games, analyzed the athletes’ pacing choice under different temperatures, humidity and sports levels, and then recommend the best pacing strategy for rowing teams of China. The model proposed in this paper complements the absence of meteorological features in the arena environment assessment and provides an algorithm basis for improving the forecast performance of pacing strategies in outdoor sports.

## 1. Introduction

The Tokyo Olympic Games, originally scheduled for 2020, were postponed by a year to 2021 due to the novel coronavirus (COVID-19) worldwide. With the postponement of major international events, the traditional training cycle of athletes was destroyed. A too-long training time makes their training load heavier, which may make it tough to reach the best condition in the competition. Once COVID-19 is brought under control, the high density of the competition schedule will further exacerbate athletes’ fatigue. Therefore, the pressure of systematic preparation for the Tokyo 2021 Olympic Games of athletes is quite severe.

In competitions, the pattern of physical load adopted by elite athletes is often the best choice made by evaluating their own abilities as well as the environment of the actual arena [1,2]. In the competitions where time is the standard of results, the load distribution in each stage is shown as the pacing strategy that athletes take [3]. Accordingly, considering vital factors inside the competition as much as possible, which is conducive to promoting the accuracy of the forecast model that helps make the match strategy, is essential for athletes to win the initiative, especially for those from nonhost countries.

In previous research, the meteorology of the arena was often ignored [4]. However, in outdoor sports events, such as rowing, competition results can be affected by altitude, temperature, humidity and wind direction easily. This mean that not thinking over meteorological information will limit the athletes’ ability to make full preparation to take the best strategy in competitions. As some variables will occur in the Tokyo 2021 Olympic Games due to the spread of COVID-19, on this account, considering the meteorological factors into the choices of tactics is significant for China’s rowing teams.

In the field of competitive sports, physical and technical responses are dependent on athletes’ strategies directly [5], which gains more and more concern in research. According to the differences in methodology, the current research on pacing strategy can be divided into two categories: the post-test pacing analysis, which refers to inversely inferring the pacing strategy based on the athlete’s performance data; and the a priori pacing analysis, which refers to the evaluation after the athlete takes multiple pacing strategies in the given laboratory conditions. This article belongs to the former. In the post-test pacing analysis, the preliminary research focused on the analysis of the pacing characteristics of top athletes [6,7], analysis of the group pacing characteristics of athletes [8,9,10,11], comparative analysis of pacing characteristics of domestic and foreign athletes [12,13,14,15,16,17], analysis of competition strategy and race pacing [18,19,20], analysis of technical movement and race pacing [21,22,23]. Above is mainly based on analyzing pacing data merely, including analysis for different sports levels [24,25,26], and different race rounds and boat types [24,27,28].

In analyzing the physical load of rowing, physiological and biomechanical data are often used to explain the differences in sports performance [29,30]. In rowing, we can often see that the athletes’ most effective distribution of effort shows a high–low–high pattern [25], and the corresponding pacing strategy is parabolic [25,26,27,28], that is, in a 2 km race (divided into 4 stages per 500 m), the speed increases rapidly to the peak in the first stage, then decreases and remains flat in the second and the third stage, and returns to a high level in the fourth stage. This is because athletes need to allocate their physical fitness reasonably during the competition, avoiding premature exertion leading to insufficient physical fitness in the later stages and drastically slowing down [31], so it shows the higher effort of the athlete in the first and last stage [32]. At this time, the impact of the difference between physiology and biomechanics on sports performance reflects the fatigue effect and fatigue-load management [33], and the choice of pacing strategies in rowing is also the decision-making process of team events.

External environmental factors, which also exert a particular influence on match load, have received attention recently in studies of pacing strategy. For example, the speeds of skiers on the uphill, downhill and flat ground are different [1], and the effort distribution in these terrains affects the skier’s overall performance [34,35,36]. In the analyses of the pacing strategy of two marathon runners, who were the best two in the world in 2013, Angus considered the influence mechanism of wind speed during the competition and the slope of the competition field on their performance [2]. However, containing a small sample of the two athletes, that research only did correlation analysis based on least-squares constraint. Therefore, massive data and systematic research on meteorology’s influence on sports are our urgent scholarly pursuit.

At the Tokyo 2021 Olympic Games, China won the qualifications of six boat types: W1x, W2-, W2x, M2x, W4x and M4x. Moreover, at the 2019 World Championships, the Chinese rowing team also won three gold medals, two of which are the W4x and M2x. Therefore, at this time China’s rowing team will be a strong contender for gold medals. Therefore, any factors that affect China’s rowing results in the Tokyo Olympics Games must be fully considered. Thus, it is urgent to supplement the necessary meteorological information for the selection of the pacing strategy of the Tokyo Olympic Games for Chinese athletes and to obtain the pacing strategy recommendations under different weather conditions.

Accordingly, this study selects hour-by-hour meteorological data of Tokyo from March 1979 to March 2020, and competition data for 6 boat types (W1x, W2-, W2x, M2x, W4x and M4x) in the 2010–2019 Olympic and World Championship finals to: (1) retrieve Tokyo historical weather data, including altitude, temperature, humidity and wind direction data, to predict the weather conditions during the Tokyo Olympics rowing competition; (2) analyze the characteristics of the pacing strategy of athletes at a different specific temperature, humidity and wind direction; (3) provide pacing strategy suggestions according to the characteristics of Chinese athletes.

Our marginal contribution may include: First, based on abundant historical data, this study provides an algorithmic basis for the weather forecast of outdoor competition events, fully mining the value of meteorological data. Second, this study makes up for the absence of meteorology in the analysis of influencing factors of pacing strategy, improving the data dimension of pace forecast, which promotes the accuracy of the forecast model. Third, we propose the best pacing strategy references for China’s rowing teams in the Tokyo 2021 Olympic Games, which will be conducive to optimize the pattern of physical load in the training and match.

## 2. Materials and Methods

### 2.1. Data Samples

Data samples used in this study include two parts: meteorological data and rowing data. The meteorological data are from OpenWeather (https://openweathermap.org/weather-conditions (accessed on 19 December 2021)). We collected the hourly meteorological data of Tokyo for 40 years, from March 1979 to March 2020. Specific data include average temperature, highest temperature, lowest temperature, somatosensory temperature, air pressure, humidity, wind speed, wind direction, weather category, cloud amount in this hour and precipitation in the past one hour (only a few data include precipitation; most of them are missing). In addition, historical data are in hours, which reflect the rules of weather changes throughout the day in a certain period. To cooperate with the machine learning algorithm of the weather prediction module for training, we also collected their real-time weather forecast data from professional weather websites. Long-term forecast data are from WeatherTAB (https://www.weathertab.com (accessed on 19 December 2021)), while 75-day forecast data are from China Weather (http://pc.weathercn.com (accessed on 19 December 2021)). The real-time weather forecast data can reflect the weather trends of each day in the future. Further, data from WeatherTAB and China Weather only include daily temperature and weather categories without hourly data, probability of precipitation, wind speed and direction. But as a training set, this does not affect the establishment of machine learning algorithms in our weather forecasting module.

The second part of the data sample is rowing data. Data used in the training of the machine learning algorithm of the rowing pacing strategy module are mainly based on the competition data of the 2010–2019 Olympic and World Championship finals provided by the official website of the World Rowing Federation (www.worldrowing.com (accessed on 19 December 2021)), including the 50 m speed and paddle frequency of the players in each game. At the same time, in the process of constructing a machine learning algorithm for the rowing pacing strategy module, we added hourly live weather information of the race location collected from OpenWeather.

### 2.2. Model Design

#### 2.2.1. Module of Weather Forecast

In the module of weather forecast, we process the data through the following three steps:

##### Data Preprocessing

For hourly meteorological data, we used the min–max normalization method. At the same time, for missing data of the past hour precipitation, we used the average value under this weather type to fit in as the precipitation at that moment. In terms of wind speed and wind direction, to better describe the feature of wind direction, but also to maintain independence between different features, we used the method of decomposing vectors in east-west, north-south directions, transforming wind speed and wind direction into vector projection values in two directions as feature input. Accordingly, we obtained 10-dimensional weather features for each hour (the 10-dimensional weather features were average temperature, highest temperature, lowest temperature, somatosensory temperature, air pressure, humidity, and wind speed in the east-west direction, wind speed in the north-south direction, cloud amount and precipitation in the past one hour. The weather category was not included because it is a feature of the tag type. It would cause this part of the dimension to be too high and decrease the weight of other features when included).

##### Generation Strategy of Training Sample

The real-time weather information of the game day and 15 days before and after it (in total 31 days) in the historical year, with the weather forecast information of that day, are what we decided to use in our model to predict the hourly highest temperature, lowest temperature, probability of precipitation, wind direction and wind speed on the game day. When generating training samples, for one part, we took a certain day in each historical year and 15 days before and after it as a pair (a pair), then generated a sample. For example, if 16 August 2018 was considered the date of the weather to be predicted, the combination of 1–31 August 2017 was a training sample, and the combination of 1–31 August 2016 was another. The other part of the training samples was the weather data of that certain day. Here we aggregated the hourly actual weather data, where we added a Gaussian noise perturbation (the perturbation parameter depends on the quality of the forecast), as the generated weather forecast feature. Specifically, in the weather forecast part based on WeatherTAB, the daily data included three-dimensional characteristics: the highest temperature, the lowest temperature and the probability of precipitation. The standard deviations of the Gaussian distribution of the three were taken as 2, 2, and 0.05, respectively; in the weather forecast part based on China Meteorology, the daily data included 10-dimensional characteristics: the maximum temperature, minimum temperature, precipitation probability, east-west wind speed, and north-south wind speed during the day and night. The standard deviations of the 5 types of Gaussian distributions were taken as 1, 1, 0.03, 0.5, and 0.5, respectively. Combining the above two parts, we obtained the samples needed for training.

##### Model Training

**Figure 1:**
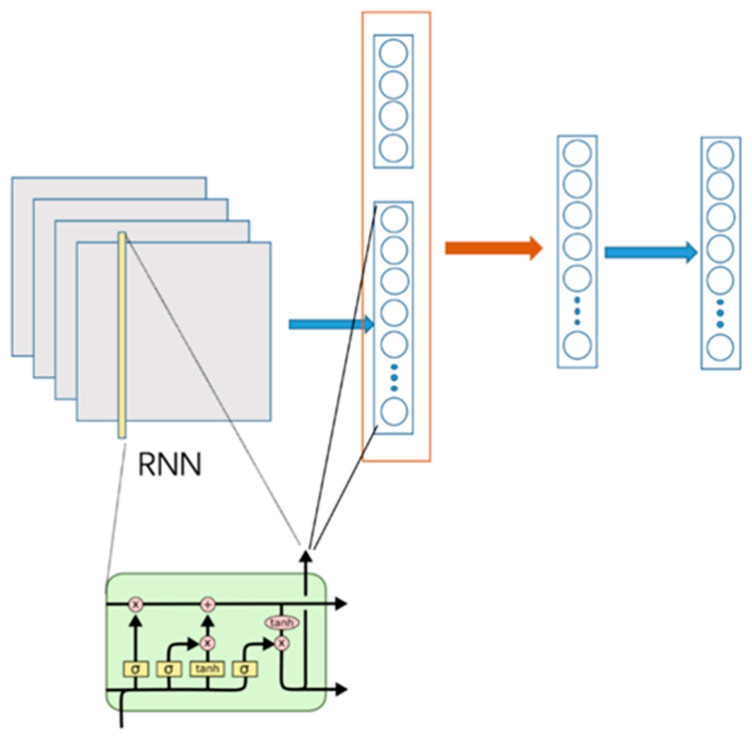
Schematic diagram of model of the weather forecast module.

Considering the potential timing laws in weather changes, we adopted the RNN model as the training model, whose schematic diagram is shown in Figure 1. The module on the left is a standard RNN structure, and the training data at each time t correspond to the 10-dimensional weather characteristics of each hour. T, which means the total number of times, has a count of 744. The output of this model and that of the weather forecast were spliced together as the input of the subsequent multilayer perceptron. After this part of the features passed through a hidden layer, we processed its output through Softmax to obtain the hour-by-hour weather forecast results within a day. It should be noted that the loss function we adopted was MSE.

Assuming that the input is $x=\left[x_{1},x_{2},\ldots ,x_{744}\right]⋃x_{f}$, in the process of this network, the calculation formula of forward is shown in Equation (1):

(1) $$\begin{array}{l} i_{t}=\sigma \left(W_{i}\cdot \left[h_{t-1},x_{t}\right]+b_{i}\right)\\ f_{t}=\sigma \left(W_{f}\cdot \left[h_{t-1},x_{t}\right]+b_{f}\right)\\ {\tilde{C}}_{t}=\tanh\left(W_{f}\cdot \left[h_{t-1},x_{t}\right]+b_{f}\right)\\ o_{t}=\sigma \left(W_{o}\cdot \left[h_{t-1},x_{t}\right]+b_{o}\right)\\ C_{t}=f_{t}\cdot C_{t-1}+i_{t}\cdot {\tilde{C}}_{t}\\ h_{t}=o_{t}\cdot \tanh\left(C_{t}\right)\\ H_{1}=\sigma \left(W_{H1}\cdot \left[h_{T},x_{f}\right]+b_{H1}\right)\\ y=soft\max\left(W_{y}H_{1}+b_{y}\right) \end{array}$$

In Equation (1), y represents the final output of the model, which is an aggregation of the output weather of different historical years and different weather forecast sources. Those were the weather conditions we were required to predict.

#### 2.2.2. Module of Rowing Pacing Strategy Forecast

In the module of rowing pacing strategy, we processed the data through the following three steps:

##### Data Preprocessing

Since the original data set did not contain the specific time of each game (not accurate to the hour but the day), we first collected and supplemented those data and matched them with the information provided by the World Rowing Federation Match. Meanwhile, except for 2019 the ranking of each team was known directly, so we used the speed information to simulate the game process and obtained the expected ranking results for other years.

##### Data Analysis

In this part of the data analysis, we divided the entire race (2000 m) into four segments: 0–500 m (Q1), 500–1000 m (Q2), 1000–1500 m (Q3) and 1500–2000 m (Q4), we could get the average speed and average paddle frequency in each segment, and then we hoped to analyze the differences of pacing strategies in different meteorological characteristics (altitude, temperature, air pressure, humidity and wind speed), different track numbers, different boat types, and athletes of different levels. Specifically, we mainly made comparisons of the average speed and paddle frequency with Q1–Q2, Q2–Q3, and Q3–Q4, and explored whether certain features affect them. In the analysis of continuous features (such as meteorological features), we calculated the correlation coefficient and the fitting slope between response variables and each explanatory variable, then judged whether this positive (negative) correlation was significant or not. Because the features belong to the order type (such as athletes’ levels) and those belong to the character type (such as boat types, track numbers), we sequentially compared the differences between every two feature values through the pairwise algorithm, and determined whether the differences were significant or not by the t-test.

##### Model Training

In addition to data analysis, we also needed to portray a good pacing strategy. Specifically, we hoped to recommend the most appropriate pacing strategy based on the given weather characteristics, track numbers and boat types through modeling. We also compared this with the benchmark model (that is, simply adopt the linear fitting method to find the strategy) to analyze the performance of the neural network model we built.

**Figure 2:**
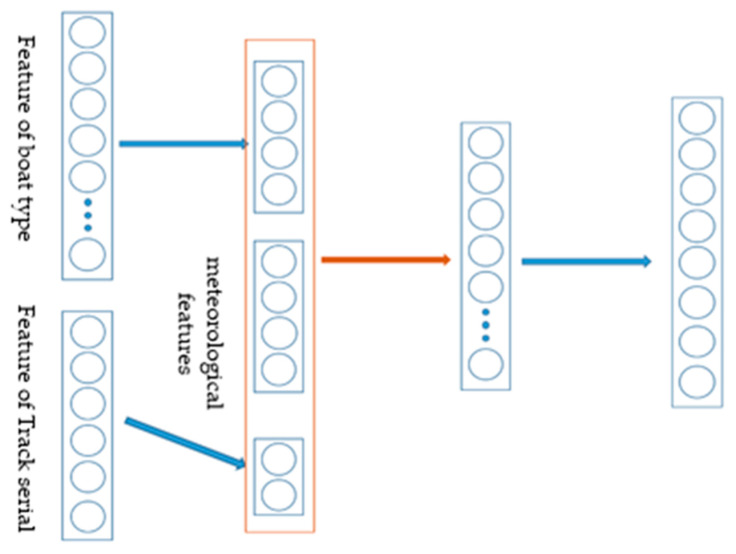
Schematic diagram of the model of the rowing pacing strategy module.

The neural network model we adopted is a multilayer perceptron; all features include altitude, temperature, air pressure, humidity, wind speed, track number and boat type. Among these, the latter two features belong to the noun category and need to be one-hot encoded. To avoid too many dimensions after encoding, which may dilute the weight of the meteorological features, we first added the network layer to these two groups of codes respectively to reduce their dimensionality. After that, the output nodes of the two networks and meteorological features were spliced together as input to the main network, and a hidden layer was inserted in the middle, after which we could obtain the output of our model. The specific network structure diagram is shown in Figure 2.

We assumed that the input is $x=\left[x_{1},x_{2},x_{3}\right]$, among which $x_{1},x_{2},x_{3},$ respectively, represent the features of the boat type, the track number, and meteorology and altitude. In this network, the forward calculation formula is as shown in Equation (2):

(2) $$\begin{array}{l} {\tilde{x}}_{1}=\sigma_{1}\left(W_{1}\cdot Onehot\left(x_{1}\right)+b_{1}\right)\\ {\tilde{x}}_{2}=\sigma_{2}\left(W_{2}\cdot Onehot\left(x_{2}\right)+b_{2}\right)\\ h_{1}=\sigma_{3}\left(W_{3}\cdot \left[{\tilde{x}}_{1},{\tilde{x}}_{2},x_{3}\right]+b_{3}\right)\\ y=W_{y}h_{1}+b_{y} \end{array}$$

where y in Equation (2) is the pacing strategy we find. In order to take advantage of the strategy of higher-level players, while considering the strategy of lower-level players, we adopted the weight sampling to prioritize the higher-ranked teams. Specifically, the first, second, and third places in the finals/semifinals, and the first and second places in other competitions can enjoy weights of 8:5:3:2:1, respectively. For the loss function, we also adopted MSE in order to keep the model’s prediction as accurate as possible in each stage, including average speed and average paddle frequency.

## 3. Results

**Figure 3:**
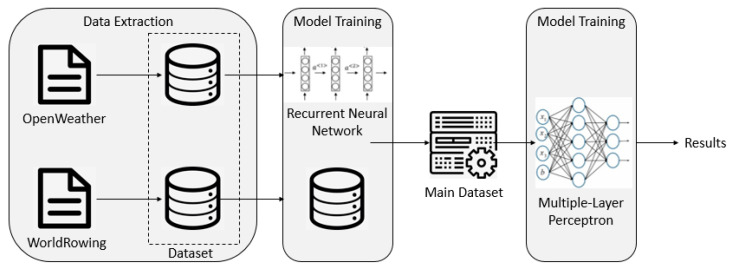
Methodology figure workflow.

Based on this, we developed a recommendation system for the rowing pacing strategy. Just input the race date and time, race location, track number and boat type, and that will provide the weather forecast results of the selected moment (including the highest temperature, the lowest temperature, the probability of precipitation, wind speed and direction), as well as the recommended pacing strategy (speed and paddle frequency) for each segment of Q1–Q4. Following the visual way of presenting the methodology in Garnica-Caparrós and Memmert (2021) [37], the methodology figure workflow is presented in Figure 3. After more than 10 epoch training, our model can be applied to the actual weather forecast.

### 3.1. The Influence of Different Meteorological Features on the Pacing Strategy

We adopted linear fitting for modeling. In terms of meteorological features, we selected altitude, temperature, air pressure, humidity and wind speed for analysis. The fitted dependent variables are in six dimensions, which include the average speed difference and the paddle frequency difference of Q1–Q2, Q2–Q3, and Q3–Q4. The model formula is shown in Equation (3):

(3) $$y=\alpha_{1}x_{1}+\alpha_{2}x_{2}+\alpha_{3}x_{3}+\alpha_{4}x_{4}+\alpha_{5}x_{5}+b$$

In order to ensure the rationality in the subsequent analysis, we only selected the top three players in all finals and semifinals as research targets, excluding athletes at the other levels. In sports competitions, we pay more attention to the results of the top three and the bottom three players. The players in other levels usually choose the follow strategy. Therefore, it is enough to cover the distribution we wanted to analyze, and the probability is approximately correct. For more details, please see the Data Availability Statement.

In the subsequent analysis, we regarded *p*-value ≥ 0.95, 0.99, and 0.999 as the first, second and third levels of significance, which are represented by *, **, and *** respectively. Correlation categories included the positive one and the negative one, which are represented by +/−, respectively, and the correlation coefficient is the fitting parameter value α. Specifically, the analysis results are as follows.

#### 3.1.1. When the Dependent Variable Is Average Speed Difference of Q1–Q2

The coefficient of goodness of fit (the proportion of the amount of information in the linear fitting to the total amount of information) $R^{2}=0.259$. The specific performance of each feature is shown in Table 1.

In Table 1, the value of P > |*t*| corresponds to 1−*p*-value, [0.025 and 0.975] representing the confidence intervals of the coefficients within this confidence range. The following tables are the same as this.

#### 3.1.2. When the Dependent Variable Is Average Speed Difference of Q2–Q3

The coefficient of goodness of fit $R^{2}=0.012$. The specific performance of each feature is shown in Table 2.

#### 3.1.3. When the Dependent Variable Is Average Speed Difference of Q3–Q4

The coefficient of goodness of fit $R^{2}=0.019$. The specific performance of each feature is shown in Table 3.

#### 3.1.4. When the Dependent Variable Is Average Paddle Frequency Difference of Q1–Q2

The coefficient of goodness of fit $R^{2}=0.088$. The specific performance of each feature is shown in Table 4.

#### 3.1.5. When the Dependent Variable Is Average Paddle Frequency Difference of Q2–Q3

The coefficient of goodness of fit $R^{2}=0.019$. The specific performance of each feature is shown in Table 5.

#### 3.1.6. When the Dependent Variable Is Average Paddle Frequency Difference of Q3–Q4

The coefficient of goodness of fit $R^{2}=0.007$. The specific performance of each feature is shown in Table 6.

### 3.2. The Influence of Track Numbers on Pacing Strategy

Here we also selected the semifinals and finals for analysis. Specifically, we used the *t*-test method of the pairwise algorithm to analyze differences in the pacing strategies of players in different tracks. In the table below, the upper part of the table is the difference significance rating of the paddle frequency data. The +/− at (track *i*, track *j*) indicates that the average paddle frequency of the players on track is significantly greater than / less than track *j*. The following stars indicate the significance level, which is consistent with the last part. The lower half of the table is the difference significance rating of the average speed data, and the meaning of the symbols is consistent with the upper part. The basis for dividing the upper and lower parts is that the effective amount of information can be represented by half a table. For example, − (**) at (3,6) indicates that the paddle frequency of the players on the third track is significantly less than the sixth track. We can directly use + (**) to represent the significant information of the paddle frequency data at (6,3). In this way, we could directly merge the two tables into one table.

#### 3.2.1. Comparison of Q1–Q2

The comparison of the influence of track numbers on pacing strategy between Q1 and Q2 is shown in Table 7.

#### 3.2.2. Comparison of Q2–Q3

The comparison of the influence of track numbers on pacing strategy between Q2 and Q3 is shown in Table 8.

#### 3.2.3. Comparison of Q3–Q4

The comparison of the influence of track numbers on pacing strategy between Q3 and Q4 is shown in Table 9.

### 3.3. The Influence of Boat Types on Pacing Strategy

Here, we selected race boats that appear more frequently in the data (including the race data of the top three in the semifinals/finals) to conduct a significant analysis of the difference in pacing strategies between every two pairs (similarly, we only considered the pacing strategies of the top three teams in the semifinals and finals). The selected boat types count as 22. The method adopted was a *t*-test, and the difference of pacing strategies between different boat types was obtained as shown in the following tables.

Notes: Due to the name of the boat type being too long, we used the numbers 1–22 in the table to mark them, and the numbers 1–22 correspond to Lightweight Men’s Double Sculls, Men’s Double Sculls, Lightweight Men’s Four, Men’s Single Sculls, Women’s Pair, Men’s Four, Men’s Quadruple Sculls, Men’s Eight, Men’s Pair, Women’s Single Sculls, Women’s Quadruple Sculls, Lightweight Women’s Double Sculls, Women’s Eight, Women’s Double Sculls, Lightweight Men’s Pair, Lightweight Women’s Single Sculls, Women’s Four, Lightweight Men’s Single Sculls, Lightweight Men’s Quadruple Sculls, Men’s Coxed Pair, PR1 Men’s Single Sculls, PR3 Mixed Coxed Four.

#### 3.3.1. Comparison of Q1–Q2

The comparison of the influence of boat types on pacing strategy between Q1 and Q2 is shown in Table 10.

In Table 10, blue means negative while green means positive. The intensity of the color is proportional to the correlation coefficient. That is, the more significant the correlation is, the darker the color is. Table 11 and Table 12 are presented in the same way.

#### 3.3.2. Comparison of Q2–Q3

The comparison of the influence of boat types on pacing strategy between Q2 and Q3 is shown in Table 11.

#### 3.3.3. Comparison of Q3–Q4

The comparison of the influence of boat types on pacing strategy between Q3 and Q4 is shown in Table 12.

### 3.4. The Influence of Player Levels on Pacing Strategy

Regarding the level of players, we divided them into four groups: the top three players in the semifinal/final (F/SF123), the last three players in the semifinal/final (F/SF456), the top three players in other competitions (PT123), and the last three players in other competitions (PT456). We conducted a comparative analysis for them. The method we adopted was the same as that of boat types. Correspondingly, we could get the differences in pacing strategies between players of different levels, which are shown in the following tables.

#### 3.4.1. Comparison of Q1–Q2

The comparison of the influence of player levels on pacing strategy between Q1 and Q2 is shown in Table 13.

#### 3.4.2. Comparison of Q2–Q3

The comparison of the influence of player levels on pacing strategy between Q2 and Q3 is shown in Table 14.

#### 3.4.3. Comparison of Q3–Q4

The comparison of the influence of player levels on pacing strategy between Q3 and Q4 is shown in Table 15.

### 3.5. Model Prediction Results

After 10 epochs of training, we established the final network model of prediction (on the given training data set, the average MSE of the sample can be as low as about 0.005). For example, we input the situation that needs to be predicted into the model: in the Men’s Four group competition, the altitude is 100 m, the temperature is 20 degrees Celsius, the air pressure is 1000 kpa, the humidity is 60%, the wind speed is 3 m/s, and track number is 3. The pacing strategy that the system returned was [5.721578 5.5232887 5.4479446 5.551791 42.585205 38.44623 38.342327 39.731567].

The returned pacing strategy had a vector of length 8. From front to back were the average speeds of Q1, Q2, Q3, and Q4, and the average paddle frequencies of Q1, Q2, Q3, and Q4. It can be seen that the average speed and average paddle frequency output by our model showed a high–low–high pattern under this particular meteorological condition, which basically agrees with the parabolic pacing strategy proposed by previous scholars [25,26,27,28,38] and reverifies the rationality of this kind of load distribution mode. Besides that, we further provided a practical reference for rowing athletes to make accurate pacing adjustment according to meteorology changes before racing.

## 4. Conclusions and Recommendations

Athletes often adjust their patterns of load based on the actual arena environment. This study aimed to get more features from data of different dimensions to simulate the competition situations, improving the forecast accuracy of pacing strategy. For long-term outdoor events such as rowing, meteorological factors are very influential for physical load, without which it is difficult to get good forecast results for the athlete. Therefore, we explored the pacing strategies adopted in the world’s top competitions in recent years and incorporated meteorology forecast results into them, so as to provide advice for China’s rowing teams about load management in the coming games. The following are our recommendations in five areas.

Strengthen the targeted training of pacing strategy during the sprint stage of Olympic preparations. In the Tokyo Olympic Games Final, on the basis of ensuring an absolutely high average speed of China’s 6 rowing teams, refer to the pacing strategy of top international rowing events in the past 10 years, and get proper training for that in the final preparation.

Achieve the optimal combination of paddle frequency, amplitude, efficiency and physiological indicators. Under the joint constraints of paddle frequency, amplitude, efficiency and physiological indicators, increase to 1–1.5 paddles/minute in each stage of the competition as much as possible. Accordingly, our rowers are required to extend the training of technique, strength, and speed daily. For instance, practice starting sail for different distances on the water, as well as catching the water for the first step after the oar enters the water. In addition, in the practice of returning the paddle on the water and on the dynamometer, attention that the key of the paddle frequency is grasping under the water and the sequence of returning the paddle is fast.

Establish a segment timing database with a 2.5% segment ratio. It is recommended to establish a segment timing database based on the 2.5% segmentation standard, reducing the segment ratio from 25% to 2.5%. Keep the tracking system in a continuous upgrade to collect more refined game data results, which helps analyze different groups’ differences in smaller segments (such as start and sprint).

Choose a training field with a climate similar to Tokyo to prepare for the Olympics. Affected by COVID-19, the 2020 Tokyo Olympic Games was postponed. Therefore, the probability of precipitation, the maximum and the minimum temperature would be expected to change greatly, even in the same period of 2021 and 2020. As a consequence, firstly, the rowing team of China should choose a proper training field, which is closer to the climatic conditions during the Tokyo competition in the final preparation. It is worth noting that the variance of the highest temperature and the lowest temperature reduced by 70.31% and 35.80% from 23 July to 8 August 2021, compared with the original game time. Secondly, it is necessary to emphasize preparing the preventive pacing strategies for large fluctuations in the probability of precipitation, the variance of which will increase by 13.39% from 23 July to 8 August 2021, compared to the original game time.

Improve the scientific level of research on physical load in China comprehensively. First, take the influence of multidimensional factors on athletes’ performance into account and shift the research of training load from an ideal environment to a more actual scene. Second, in addition to regression analysis and variance analysis, it is recommended to introduce interdisciplinary research methods such as least-squares constraints, KS normal distribution test, optimization model, and machine learning. Third, it is suggested to combine wearable devices, computer vision, and the Internet of Things (IoT) in data collection, by which more fine-grained data and more types of data (such as physiological data, biomechanical data, field environment data, etc.) can be obtained. Fourth, physiological and psychological data such as heart rate, oxygen uptake, and RPE can be introduced into the internal load analysis.

## Figures and Tables

**Table 1:** The correlation performance of each feature when the dependent variable is average speed difference of Q1–Q2.

	Correlation Coefficient	Coefficient Standard Deviation	*t*	P > |*t*|	[0.025	0.975]	Correlation Categories ^1^	Significance Level
altitude	0.0002	0.0000	7.0990	0.0000	0.0000	0.0000	+	***
temperature	−0.1110	0.0010	−14.3390	0.0000	−0.0130	−0.0090	−	***
air pressure	0.0004	0.0010	0.7220	0.4700	−0.0010	0.0020		
humidity	0.0011	0.0000	5.4640	0.0000	0.0010	0.0010	+	***
wind speed	0.0171	0.0020	8.9490	0.0000	0.0130	0.0210	+	***

^1^ − means negative correlation, + means positive correlation. *** means significance at level of 1%.

**Table 2:** The correlation performance of each feature when the dependent variable is average speed difference of Q2–Q3.

	Correlation Coefficient	Coefficient Standard Deviation	*t*	P > |*t*|	[0.025	0.975]	Correlation Categories ^1^	Significance Level
altitude	0.0000	0.0000	−2.6340	0.0090	−0.0001	0.0000	−	**
temperature	0.0004	0.0000	0.9620	0.3360	0.0000	0.0010		
air pressure	0.0009	0.0000	2.6800	0.0070	0.0000	0.0020	+	**
humidity	−0.0003	0.0000	−2.2090	0.0270	0.0000	0.0000	−	*
wind speed	−0.0033	0.0010	−3.0270	0.0030	−0.0060	−0.0010	−	**

^1^ − means negative correlation, + means positive correlation. * and ** mean significance at level of 10%, 5% respectively.

**Table 3:** The correlation performance of each feature when the dependent variable is average speed difference of Q3–Q4.

	Correlation Coefficient	Coefficient Standard Deviation	*t*	P > |*t*|	[0.025	0.975]	Correlation Categories ^1^	Significance Level
altitude	0.0000	0.0000	0.3340	0.7380	0.0000	0.0000		
temperature	−0.0034	0.0010	−4.9570	0.0000	−0.0050	−0.0020	−	***
air pressure	−0.0003	0.0010	−0.5620	0.5740	−0.0010	0.0010		
humidity	−0.0005	0.0000	−2.8920	0.0040	−0.0010	0.0000	−	**
wind speed	−0.0013	0.0020	−0.7420	0.4580	−0.0050	0.0020		

^1^ − means negative correlation. ** and *** mean significance at level of 5% and 1% respectively.

**Table 4:** The correlation performance of each feature when the dependent variable is average paddle frequency difference of Q1–Q2.

	Correlation Coefficient	Coefficient Standard Deviation	*t*	P > |*t*|	[0.025	0.975]	Correlation Categories ^1^	Significance Level
altitude	0.0036	0.0000	12.5810	0.0000	0.0030	0.0040	+	***
temperature	0.0365	0.0090	4.0590	0.0000	0.0190	0.0540	+	***
air pressure	0.0045	0.0070	0.6810	0.4960	−0.0090	0.0180		
humidity	0.0121	0.0020	5.2150	0.0000	0.0080	0.0170	+	***
wind speed	0.1405	0.0220	6.2910	0.0000	0.0970	0.1840	+	***

^1^ + means positive correlation. *** means significance at level of 1%.

**Table 5:** The correlation performance of each feature when the dependent variable is average paddle frequency difference of Q2–Q3.

	Correlation Coefficient	Coefficient Standard Deviation	*t*	P > |*t*|	[0.025	0.975]	Correlation Categories ^1^	Significance Level
altitude	−0.0007	0.0000	−4.8490	0.0000	−0.0010	0.0000	−	***
temperature	0.0012	0.0050	0.2480	0.8040	−0.0080	0.0100		
air pressure	0.0022	0.0030	0.6350	0.5250	−0.0050	0.0090		
humidity	−0.0034	0.0010	−2.8010	0.0050	−0.0060	−0.0010	−	**
wind speed	−0.0103	0.0120	−0.8880	0.3740	−0.0330	0.0120		

^1^ − means negative correlation. ** and *** mean significance at level of 5% and 1% respectively.

**Table 6:** The correlation performance of each feature when the dependent variable is average paddle frequency difference of Q3–Q4.

	Correlation Coefficient	Coefficient Standard Deviation	*t*	P > |*t*|	[0.025	0.975]	Correlation Categories	Significance Level
altitude	0.0003	0.0000	1.2010	0.2300	0.0000	0.0010		
temperature	−0.0105	0.0090	−1.2060	0.2280	−0.0280	0.0070		
air pressure	0.0114	0.0060	1.7800	0.0750	−0.0010	0.0240		
humidity	0.0008	0.0020	0.3390	0.7350	−0.0040	0.0050		
wind speed	−0.0185	0.0220	−0.8560	0.3920	−0.0610	0.0240		

**Table 7:** The comparison of track numbers’ influence on paddle frequency between Q1 and Q2.

	Track 1 ^1^	Track 2	Track 3	Track 4	Track 5	Track 6
**Track 1**	/		+ (*)			
**Track 2**		/	+ (*)			
**Track 3**			/			+ (**)
**Track 4**				/		
**Track 5**		− (**)		+ (*)	/	+ (*)
**Track 6**	− (***)	− (***)	− (***)	− (***)	− (***)	/

^1^ − means negative correlation, + means positive correlation. *, ** and *** mean significance at level of 10%, 5% and 1% respectively.

**Table 8:** The comparison of track numbers’ influence on paddle frequency between Q2 and Q3.

	Track 1 ^1^	Track 2	Track 3	Track 4	Track 5	Track 6
**Track 1**	/	− (***)	− (*)	− (**)	− (**)	
**Track 2**	+ (***)	/	+ (*)	+ (*)	+ (*)	+ (***)
**Track 3**	+ (***)		/			+ (**)
**Track 4**	+ (***)			/		+ (***)
**Track 5**	+ (***)				/	+ (**)
**Track 6**		− (***)	− (***)	− (***)	− (***)	/

^1^ − means negative correlation, + means positive correlation. *, ** and *** mean significance at level of 10%, 5% and 1% respectively.

**Table 9:** The comparison of track numbers’ influence on paddle frequency between Q3 and Q4.

	Track 1 ^1^	Track 2	Track 3	Track 4	Track 5	Track 6
**Track 1**	/	− (**)	− (***)	− (***)	− (***)	
**Track 2**	+ (**)	/	− (*)		+ (*)	+ (***)
**Track 3**	+ (**)		/	+ (*)		+ (***)
**Track 4**	+ (***)			/		+ (***)
**Track 5**	+ (*)			− (*)	/	+ (***)
**Track 6**		− (***)	− (***)	− (***)	− (***)	/

^1^ − means negative correlation, + means positive correlation. *, ** and *** mean significance at level of 10%, 5% and 1% respectively.

**Table 10:** The comparison of boat types’ influence on pacing strategy between Q1 and Q2.

	1 ^1^	2	3	4	5	6	7	8	9	10	11	12	13	14	15	16	17	18	19	20	21	22
**1**	/							− (*)						(*)		+ (*)						
**2**	− (*)	/				+ (*)		− (*)						− (*)		+ (*)			+ (*)			
**3**	+ (***)	+ (***)	/																			
**4**		+ (*)	− (***)	/		+ (*)									+ (*)	+ (**)			+ (*)			
**5**	− (**)		− (***)	− (**)	/	+ (*)		− (*)						− (*)	+ (*)	+ (**)			+ (**)			
**6**	− (**)		− (***)	− (**)		/	− (*)	− (**)	− (*)	− (**)	− (*)	− (*)		− (***)				− (*)				− (*)
**7**	+ (*)	+ (***)	− (***)	+ (*)	+ (***)	+ (***)	/								+ (*)	+ (**)			+ (*)			
**8**		+ (**)	− (***)		+ (***)	+ (**)		/		+ (*)		+ (**)			+ (**)	+ (***)		+ (*)	+ (**)			
**9**	− (*)		− (***)	− (*)			− (***)	− (**)	/							+ (**)			+ (*)			
**10**		+ (**)	− (***)		+ (***)	+ (***)			+ (***)	/					+ (*)	+ (**)			+ (**)			
**11**			− (***)		+ (*)		− (*)				/				+ (*)	+ (**)			+ (**)			
**12**		+(***)	− (***)		+(***)	+(***)			+(***)		+(*)	/		− (**)	+(*)	+(**)			+(**)			− (*)
**13**			− (***)		+(**)	+(*)			+(*)				/		+(*)	+(*)			+(**)			
**14**			− (***)		+(**)	+(**)	− (*)		+(*)			− (*)		/	+(***)	+(***)	+(*)		+(***)		+(*)	
**15**			− (***)		+(**)	+(**)			+(*)						/		− (*)	− (*)				− (**)
**16**	+(*)	+(***)	− (***)	+(*)	+(***)	+(***)			+(***)		+(**)			+(**)	+(*)	/	− (*)	− (**)		− (*)		− (**)
**17**	− (***)	− (***)	− (***)	− (***)	− (**)	− (*)	− (***)	− (***)	− (***)	− (***)	− (***)	− (***)	− (***)	− (***)	− (***)	− (***)	/		+(**)			
**18**		+(**)	− (***)		+(***)	+(***)			+(**)							− (*)	+(***)	/	+(**)			
**19**			− (***)				− (*)			− (*)		− (*)				− (**)	+(***)		/	− (**)		− (***)
**20**		+(*)	− (***)		+(***)	+(**)			+(**)		+(*)			+(*)			+(***)		+(*)	/		
**21**	− (**)		− (***)	− (**)			− (***)	− (***)		− (***)	− (**)	− (***)	− (***)	− (***)	− (***)	− (***)	+(**)	− (***)	− (*)	− (***)	/	
**22**	− (***)	− (**)	− (***)	− (***)	− (**)	− (*)	− (***)	− (***)	− (**)	− (***)	− (***)	− (***)	− (***)	− (***)	− (***)	− (***)		− (***)	− (***)	− (***)	− (***)	/

^1^ − means negative correlation, + means positive correlation. *, ** and *** mean significance at level of 10%, 5% and 1% respectively.

**Table 11:** The comparison of boat types’ influence on pacing strategy between Q2 and Q3.

	1 ^1^	2	3	4	5	6	7	8	9	10	11	12	13	14	15	16	17	18	19	20	21	22
**1**	/						− (*)											− (**)			+ (*)	
**2**		/						+ (*)					+ (*)			+ (**)					+ (**)	+ (*)
**3**		+ (*)	/				− (**)											− (**)			+ (*)	
**4**				/			− (*)											− (**)			+ (*)	
**5**	+ (**)	+ (**)		+ (*)	/			+ (*)								+ (*)		− (*)			+ (*)	
**6**						/		+ (*)								+ (*)		− (*)			+ (*)	+ (*)
**7**			− (*)		− (**)		/	+ (**)					+ (**)			+ (***)					+ (**)	+ (**)
**8**			− (*)	− (*)	− (***)	− (*)		/						− (**)	− (*)		− (*)	− (***)				
**9**					− (*)				/									− (*)			+ (*)	
**10**	+ (***)	+ (***)		+ (*)			+ (***)	+ (***)	+ (*)	/								− (*)			+ (*)	
**11**								+ (*)			/							− (*)				
**12**		+ (*)					+ (*)	+ (*)		− (*)		/						− (**)			+ (*)	
**13**			− (*)	− (*)	− (***)	− (*)				− (***)	− (*)	− (*)	/	+ (*)	+ (*)			− (**)				
**14**		+ (*)					+ (*)	+ (**)		− (*)			+ (**)	/		+ (**)		− (*)			+ (**)	+ (*)
**15**								+ (*)					+ (*)		/	+ (*)					+ (*)	+ (*)
**16**							+ (*)	+ (**)					+ (**)			/		− (***)				
**17**					− (*)					− (*)							/				+ (*)	+ (*)
**18**					− (*)			+ (*)		− (**)			+ (*)					/		+ (*)	+ (***)	+ (**)
**19**								+ (*)					+ (*)						/			+ (*)
**20**													+ (*)							/		
**21**					− (*)					− (*)			+ (*)								/	
**22**					− (*)					− (**)												/

^1^ − means negative correlation, + means positive correlation. *, ** and *** mean significance at level of 10%, 5% and 1% respectively.

**Table 12:** The comparison of boat types’ influence on pacing strategy between Q3 and Q4.

	1 ^1^	2	3	4	5	6	7	8	9	10	11	12	13	14	15	16	17	18	19	20	21	22
**1**	/	− (**)	− (***)	+ (**)		− (***)	− (***)	− (*)		+ (**)		− (*)					− (*)	− (*)	− (*)		+ (***)	+ (*)
**2**	+ (***)	/	− (*)	+ (***)	+ (**)	− (*)			+ (*)	+ (***)						+ (*)					+ (***)	+ (***)
**3**	+ (**)		/	+ (***)	+ (***)				+ (**)	+ (***)	+ (*)	+ (**)	+ (**)	+ (***)	+ (**)	+ (***)		+ (*)			+ (***)	+ (***)
**4**	− (***)	− (***)	− (***)	/		− (***)	− (***)	− (***)	− (**)		− (**)	− (***)	− (*)	− (***)	− (**)		− (**)	− (***)	− (**)	− (*)	+ (**)	
**5**	− (**)	− (***)	− (***)		/	− (***)	− (***)	− (**)		+ (*)	− (*)	− (**)		− (*)	− (*)		− (**)	− (**)	− (*)	− (*)	+ (***)	+ (*)
**6**	+ (***)			+ (***)	+ (***)	/			+ (***)	+ (***)	+ (*)	+ (***)	+ (*)	+ (***)	+ (*)	+ (***)		+ (*)			+ (***)	+ (***)
**7**	+ (**)			+ (***)	+ (***)	− (*)	/		+ (**)	+ (***)		+ (*)	+ (*)	+ (**)		+ (**)					+ (***)	+ (***)
**8**	+ (***)			+ (***)	+ (***)			/		+ (***)						+ (*)					+ (***)	+ (***)
**9**	+ (*)			+ (***)	+ (***)	− (**)		− (*)	/	+ (**)											+ (***)	+ (*)
**10**	− (***)	− (***)	− (***)			− (***)	− (***)	− (***)	− (***)	/	− (***)	− (***)	− (*)	− (***)	− (**)	− (*)	− (***)	− (***)	− (***)	− (**)	+ (**)	
**11**		− (*)	− (**)	+ (**)	+ (*)	− (***)	− (*)	− (**)		+ (**)	/										+ (***)	+ (**)
**12**		− (***)	− (***)	+ (***)	+ (**)	− (***)	− (***)	− (***)	− (**)	+ (***)		/				+ (*)					+ (***)	+ (***)
**13**				+ (***)	+ (***)					+ (***)		+ (*)	/								+ (***)	+ (**)
**14**		− (***)	− (***)	+ (***)	+ (*)	− (***)	− (**)	− (***)	− (*)	+ (***)			− (*)	/							+ (***)	+ (**)
**15**		− (*)	− (*)	+ (**)	+ (**)	− (**)		− (**)		+ (***)					/						+ (***)	+ (***)
**16**		− (***)	− (***)	+ (**)	+ (*)	− (***)	− (***)	− (***)	− (*)	+ (**)			− (*)			/	− (*)	− (*)			+ (***)	
**17**				+ (***)	+ (**)	− (*)		− (*)		+ (***)							/				+ (***)	+ (***)
**18**		− (***)	− (***)	+ (***)	+ (*)	− (***)	− (**)	− (***)	− (*)	+ (***)								/			+ (***)	+ (**)
**19**	+ (*)			+ (***)	+ (***)					+ (***)	+ (*)	+ (**)		+ (**)	+ (*)	+ (**)		+ (**)	/		+ (***)	+ (***)
**20**	+ (***)			+ (***)	+ (***)				+ (*)	+ (***)	+ (***)	+ (***)	+ (*)	+ (***)	+ (**)	+ (***)	+ (*)	+ (***)		/	+ (***)	+ (***)
**21**	− (***)	− (***)	− (***)		− (**)	− (***)	− (***)	− (***)	− (***)	− (*)	− (***)	− (***)	− (***)	− (***)	− (***)	− (***)	− (***)	− (***)	− (***)	− (***)	/	
**22**		− (*)	− (**)			− (**)	− (*)	− (**)					− (*)						− (**)	− (***)	+ (**)	/

^1^ − means negative correlation, + means positive correlation. *, ** and *** mean significance at level of 10%, 5% and 1% respectively.

**Table 13:** The comparison of player levels’ influence on pacing strategy between Q1 and Q2.

	F/SF123 ^1^	F/SF456	PT123	PT456
**F/SF123**	/			+ (**)
**F/SF456**		/		+ (***)
**PT123**			/	
**PT456**	− (***)	− (*)	− (**)	/

^1^ − means negative correlation, + means positive correlation. *, ** and *** mean significance at level of 10%, 5% and 1% respectively.

**Table 14:** The comparison of player levels’ influence on pacing strategy between Q2 and Q3.

	F/SF123 ^1^	F/SF456	PT123	PT456
**F/SF123**	/	+ (***)	+ (***)	+ (***)
**F/SF456**	− (***)	/		+ (***)
**PT123**	− (***)	+ (***)	/	+ (***)
**PT456**	− (***)	− (***)	− (***)	/

^1^ − means negative correlation, + means positive correlation. *** means significance at level of 1%.

**Table 15:** The comparison of player levels’ influence on pacing strategy between Q3 and Q4.

	F/SF123 ^1^	F/SF456	PT123	PT456
**F/SF123**	/	+ (***)	+ (***)	+ (***)
**F/SF456**	− (***)	/	+ (***)	+ (***)
**PT123**	− (***)	− (***)	/	+ (***)
**PT456**	− (***)	− (***)	− (**)	/

^1^ − means negative correlation, + means positive correlation. ** and *** mean significance at level of 5% and 1% respectively.

## Data Availability

The dataset generated and analyzed during the current study is available at https://github.com/CLMtsgzy/Rowing-Pacing-Strategy.git (accessed on 19 December 2021).
